# Establishment of five immortalized human ovarian surface epithelial cell lines via SV40 T antigen or HPV E6/E7 expression

**DOI:** 10.1371/journal.pone.0205297

**Published:** 2018-10-08

**Authors:** Ha-Yeon Shin, Wookyeom Yang, Eun-ju Lee, Gwan Hee Han, Hanbyoul Cho, Doo Byung Chay, Jae-hoon Kim

**Affiliations:** Gangnam Severance Hospital, Yonsei University College of Medicine, Seoul, Republic of Korea; The University of Hong Kong, HONG KONG

## Abstract

**Background:**

Human ovarian surface epithelial (HOSE) cells are a critical cell source for ovarian cancer research; however, they are difficult to obtain and maintain under standard laboratory conditions in large quantities. The aim of this study was to generate immortalized HOSE (IHOSE) cells with maintained properties to the original cell source, thereby guaranteeing a sufficiently large cell quantity for ovarian cancer research.

**Methods:**

HOSE cells isolated from four non-cancer patients and five IHOSE cell lines were established by induction of HPV-E6/E7 expression or SV40 large T antigen using a lenti-viral system. Each of IHOSE cells was confirmed to be distinct by STR profiling. RNA-sequencing was used to compare gene expression profiles in HOSE, IHOSE and ovarian cancer cells.

**Results:**

RNA-sequencing results revealed a stronger linear correlation in gene expression between IHOSE and HOSE cells (R^2^ = 0.9288) than between IHOSE or HOSE cells and ovarian cancer cells (R^2^ = 0.8562 and R^2^ = 0.7982, respectively). The gene expression pattern of 319 differentially expressed genes revealed minimal differences between HOSE and IHOSE cells, while a strong difference between ovarian cancer cells and HOSE or IHOSE cells was observed. Furthermore, the five IHOSE cell lines displayed morphological characteristics typical of epithelial cells but showed a lower level of EpCAM, CD133 and E-cadherin, as cancer stem marker, than ovarian cancer cells. Moreover, unlike cancer cells, IHOSE cells could not form colonies in the anchorage-independent soft agar growth assay.

**Conclusion:**

These findings demonstrate that five newly established IHOSE cell lines have characteristics of progenitor HOSE cells while exhibiting continuous growth, and thus, should be highly useful as control cells for ovarian cancer research.

## Introduction

Ovarian cancer has a poor prognosis with the lowest survival rate among all gynecological cancers, which is mainly due to the lack of early symptoms, resulting in diagnosis when the cancer has already progressed to an advanced stage [1]. The World Cancer Report of the International Agency for Research on Cancer stated that 114,240 women were diagnosed with ovarian cancer in 2014, with a 5-year survival rate below 45% [2]. In the United States, the mortality rate of ovarian cancer ranks fifth among all cancer patients, with 22,440 new patients with ovarian cancer diagnosed in 2017 resulting in 14,080 deaths [1]. Improvement of this situation requires more extensive research on epithelial ovarian cancer, which necessitates an adequate quantity of human ovarian surface epithelial (HOSE) cells as controls for comparisons of the specific properties and biological behaviors of ovarian cancer cells. However, HOSE cells have an extremely short life span in monolayer cell culture, which has thus far limited ovarian cancer research. Although culture of HOSE cells in a modified medium (NOSE-CM) could potentially prolong cell survival compared to culture in more common media [3], this method alone cannot sustain the amount of HOSE cells required for basic research purposes. Therefore, cell immortalization methods that allow continuous cell growth without limitation of cellular life span have been actively investigated [4–7], including viral gene induction that controls proteins involved in the cell cycle and artificial expression of core proteins related to cell immortality [8]. Specifically, immortalized cell lines are established by overexpression of the HPV-E6/E7 protein or SV40 T antigen in healthy ovarian surface epithelial cells [4, 5]. Alternatively, overexpression of human telomerase (hTERT) instead of HPV-E6/E7 has been reported to maintain cellular functions of pRB and p53 [6]. Moreover, the success rate of producing immortalized cell lines increases when hTERT overexpression is coupled with overexpression of HPV-E6/E7 or SV40 T antigen compared to overexpression of hTERT alone [7]. Furthermore, once an immortalized cell line is established, it must be verified by confirming that the characteristics of the progenitor cell line are preserved. For an epithelial cell line, such observations are based on examination of the cellular morphology and expression pattern of the epithelial marker cytokeratin [9]. In addition, any changes in chromosomes that may have been induced by the immortalization protocol are screened by karyotype analysis [10] and/or the presence of gene mutations from the progenitor cell using whole-exome sequencing [11]. Actually, ovarian cancer has been known to originate from the ovarian surface epithelium (OSE) since the mid-90s to early 2000s [12–15]. To understand the ovarian carcinogenesis, immortalized OSE (IOSE) cells were constructed by the overexpression of immortalized SV-40 T antigen, telomerase and the HPV E6/E7 protein by various study groups [12–14, 16–20]. Several studies have been attempted to identify the genetic differences and their functions in IOSE cells as an intermediate step in cancer, in order to understand the function of pre-malignant or tumorigenic cells [12–15, 17, 21]. In Clinical Cancer Research article (2003), the differences in the gene expression between the IOSE and normal OSE cells were compared using microarray, and whether IOSE cells could be used as a control for ovarian cancer research was discussed [19]. Since then, in the late 2000s and mid-2010, an IOSE cell line was used as an experimental control for ovarian cancer, and these cells were used as a tool to identify the functions and mechanisms of genes implicated in cancer cells [20, 22–28]. Although it has been concluded that the origin of serous ovarian carcinoma arises from the fallopian tube epithelium, and that the endometrioid and clear cell carcinoma are derived from endometriosis [29–31], IOSE cells are still used in many studies as an experimental control and to understand gene functions. Moreover, various kinds of immortalized cells generated in the future might serve as important experimental tools [26–28]. Here, we used an RNA sequencing technique for gene expression profiling to verify whether our established immortalized HOSE (IHOSE) cells retained characteristics of the progenitor HOSE cells in comparison to those of ovarian cancer cells. In addition, we investigated whether the IHOSE cells demonstrated any malignant features of cancer cells based on cancer stem cell marker expression and an anchorage-independent growth assay. Confirmation of these characteristics and stability of IHOSE cells established with the proposed method could provide a useful resource for comparative or gene expression studies on ovarian cancer toward identification of novel therapeutic and/or diagnostic targets.

## Materials and methods

### Cell culture

HOSE cells were obtained by scraping the surfaces of healthy ovaries from patients without cancer, and were provided by the Korea Gynecologic Cancer Bank through the Bio&Medical Technology Development Program of the Ministry of the National Research Foundation (NRF) funded by the Korea government (MSIT) (NRF-2017M3A9B8069610). The monolayer of HOSE cells was cultured with M199/MCDB basal medium (1:1) supplemented with 10% Fetal bovine serum FBS and 1% penicillin/streptomycin. HOSE cells were maintained up to six passages, but most HOSE cells aged at two or three passages. IHOSE cells were established by transfecting HPV E6/E7 and SV40 T antigen to short-cultured HOSE cells using a lentiviral system. Cells were grown in DMEM containing 10% FBS with 1% penicillin / streptomycin and cultured at 37°C in 5% CO_2_. Images of the cells were acquired using an Cell Imaging System (Thermo Fisher Scientific, Rockford, IL). This study was approved by the institutional review board of Gangnam Severance Hospital, and informed consent was obtained from each patient before sample collection. All cell lines were established in the Laboratory of Obstetrics and Gynecology, Gangnam Severance Hospital, Seoul, Korea. Five IHOSE cell lines were deposited with the Korean Gynecology Cancer Bank (KGCB), and are available to researchers. SKOV3 and OVCAR3 cell lines were purchased from the American Type Culture Collection (ATCC, Manassas, VA). SKOV3 and OVCAR3 cell line was maintained in RPMI-1640 supplemented with 1% penicillin / streptomycin and were cultured at 37°C in 5% CO_2_.

### Lentiviral production and infection

To generate pCDH-HPV-E6 and pCDH-HPV-E7, cDNA encoding HPV-E6 or HPV-E7 was amplified from total RNA of cervical cancer SiHa cells using the following primer sets for HPV-E6: 5′-AAGAATTCATGCACCAAAAGAGAACTGCAAT-3′ (forward) and 5′-AAGGATCCTTACAGCTGGGTTTCTCTACGTG-3′ (reverse), and HPV-E7: 5′-AAGAATTCATGCATGGAGATACACCTACATT-3′ (forward) and 5′-AAGGATCCTTATGGTTTCTGAGAACAGATGG-3′ (reverse). The amplified cDNA was cloned into EcoRI and BamHI restriction sites of the pCDH-EF1-MCS-T2A-copGFP Lentivector (System Biosciences, Mountain View, CA). pLenti CMV/TO SV40 small+Large T vector was obtained from Addgene (Cambridge, MA). HEK293T cells (1 × 10^6^) were co-transfected with 2 μg lentiviral vector and 2 μg pPACKH1 Lentivector Packaging Kit (System Biosciences, Palo Alto, CA). The crude viral supernatant was collected 48 h and 72 h after transfection (collected viral medium, 10 mL). HOSE cells were infected with 500 μL of the collected crude viral medium per dish. The medium with infected cells was replaced with fresh medium after 24 h. During this process, the infected cells did not have with a selectable marker, and single colonies were not selected.

### Short tandem repeat (STR) profiling and mycoplasma contamination test

Genomic DNA of IHOSE cells were extracted by Total DNA Extreaction Kit. (iNtRON Biotechnology, Seoul, Republic of Korea). STR profiling analysis was conducted through the Korea Cell Line Bank (http://cellbank.snu.ac.kr). Genomic DNA was processed for STR profiling using PCR Amplification kit (Applied Biosystems, Foster, CA) according to the manufacturer's direction. After PCR amplification, the samples were analyzed on the ABI 3530xl Genetic Analyzer (Applied Biosystems) using the GeneMapper v5.0 software (Applied Biosystems). Each sample was amplified using Mycoplasma PCR Detection kit (iNtRON Biotechnology) according to the manufacturer’s suggested protocol. The PCR products were separated in 1% agarose gel at 30 V for 30 min and detected using Gel Doc XR+ imaging system (Bio-Rad Laboratories, Inc, Hercules, CA)

### Ion AmpliSeq Transcriptome library Preparation

When 3 HOSE, 5 IHOSE and 2 ovarian cancer cells were 70% confluence, total RNA was extracted with TRIzol Reagent according to manufacturer’s protocol (Ambion, Carlsbad, CA) and were quantified using Qubit RNA HS Assay Kit (Life Technologies, Carlsbad, CA) and calculated percentage of RNA fragments larger than 200nt using smear analysis of Agilent 2100 Bioanalyzer (Agilent Technologies, Santa Clara, CA). DNA samples were quantified using Qubit dsDNA HS Assay Kit (Life Technologies). An Ion AmpliSeq Transcriptome library was constructed with the Ion Transcriptome Human Gene Expression Kit (Life Technologies) as per manufacturer’s protocol. 10 ng of total RNA were reverse transcribed to make cDNA by random priming. cDNA product was amplified target genes using the Ion AmpliSeq Human Gene Expression Core Panel with the Ion AmpliSeq Library Kit Plus. After primer digestion, adapters and molecular barcodes were ligated to the amplicons followed by magnetic bead purification. This library was amplified for a total of 5 cycles and purified. Amplicon size and DNA concentration were measured using an Agilent High Sensitivity DNA Kit (Agilent Technologies) according to the manufacturer’s recommendation.

### Ion Proton sequencing

Sample emulsion PCR, emulsion breaking, and enrichment were performed using the Ion PI Template OT2 200 Kit v3 (Life Technologies, Part #4488318 Rev. B.0), according to the manufacturer’s instructions. Multiple barcoded libraries were combined together with equal molar ratios for one Ion PI v2 chip. 2 pooled Ion AmpliSeq Exome libraries were loaded onto a single Ion PI v2 chip. 5 pooled Ion AmpliSeq Transcriptome libraries were loaded onto a single Ion PI v2 chip. Subsequent emulsion PCR and enrichment of the sequencing beads of the pooled libraries was performed using the Ion OneTouch system (Life Technologies) according to the manufacturer’s protocol within about 7 hours. Finally, 520 Flows sequencing was done on the Ion PI v2 chip using Ion PI Sequencing 200 Kit v3 (Life Technologies, Part #4488315 Rev. B.0) on the Ion Proton sequencer (Life Technologies).

### RNA sequencing read mapping and gene expression analysis

RNA sequencing reads were mapped to the human genome (hg19) and calculated the reads count for each gene. Finally, each gene was normalized using RPKM. And we analyzed scatter plot, heat-map and differentially expressed genes (DEGs). The heat map was drawn as log values. This whole process was analyzed using DNASTAR Lasergene 15 software. David bioinformatics database (https://david.ncifcrf.gov/) was used for Gene ontology analysis.

### Real-time PCR

At 70–80% of confluence, all cells were washed with PBS and total RNA was extracted with TRIzol Reagent according to manufacturer’s protocol (Ambion, Carlsbad). Total RNA (1 μg) from each sample was reverse-transcribed into cDNA using Maxima First Strand cDNA Synthesis Kit (Thermo Scientific, Waltham, MA) according to the manufacturer’s protocol. Real-time polymerase chain reaction (PCR) was performed to quantify mRNA expression using SYBR Green PCR Master Mix (Enzynomics, Daejeon, Republic of Korea) and an ABI PRISM 7300 real-time PCR system (Applied Biosystems, Foster City, CA) according to the manufacturer’s instructions. Relative mRNA expression was quantified using the comparative Ct (ΔCt) method and expressed as 2^-^ΔΔ^Ct^, where ΔΔCt = ΔE– ΔC, ΔE = CtE target–CtE GAPDH, and ΔC = CtC target–CtC GAPDH (E = experimental result and C = controls). Each assay was done in triplicate and expressed as the mean ± standard error (SE). A series of dilutions were prepared from a stock solution of total RNA to generate a standard curve to determine reaction efficiencies. The primers for PCR were as follows: HPS1: Forward 5′-CTCCAAAAGTGAGCCCGGAT-3′and Reverse 5′-ATGAGCCTCTGCACTTGGTC-3′, KRT222: Forward 5′-AAGGGGCCTTGAAAACTCCC-3′ and Reverse 5′-GGAGGTGGCGATAAGTTGCT-3′, PKP1: Forward 5′-AGGAGGAACTCATTGCCGAC-3′ and Reverse 5′-AGCTCAGGTTCCTCAAGCAG-3′, OR522N1: Forward 5′-ACTGCAAGGGCAACGTCATA-3′ and Reverse 5′-ATCAAAGCCCCCAATCAGCA-3′, and GAPDH: Forward 5′-GAAGGTGAAGGTCGGAGT-3′ and Reverse 5′-GAAGATGGTGATGGGATTTC-3′.

### Protein extraction and western blotting

Total cell lysates were isolated using cell lysis buffer (150 mM NaCl, 50 mM Tris pH 7.4, 1% NP-40, 1 mM EDTA, 1 mM sodium orthovanadate, 1 mM NaF, and 1 mM sodium pyrophosphate) containing proteinase inhibitor cocktail (Roche, Nutley, NJ). Protein concentrations were determined by BCA assay (Sigma-Aldrich, St. Louis, MO). Proteins were separated by SDS-PAGE and transferred from gels to 0.2 μm nitrocellulose membranes (Pall Corporation, Washington, NY). Protein bands were visualized using western blotting luminol reagent (Santa Cruz Biotechology, Inc., Dallas, Texas) after binding with a HRP-conjugated secondary antibody. Anti-Cytokeratin 7 (sc-23879), anti-Cytokeratin 18 (sc-515852), anti-EpCAM (sc-25308), anti-α-Actinin (sc-17829), and anti-GAPDH (sc-59541) antibodies were obtained from Santa Cruz Biotechnology, while anti-E-cadherin (#14472), anti-CD133 (#5860), and anti-CD44 (#3570) antibodies were purchased from Cell Signaling Technology (Danvers, MA)

### Cell proliferation assay

To directly count the number of cells, cells were seeded in a 6-well plate at a density of 4 × 10^4^ cells per well and cultured for 8 days. Ten microliters of resuspended cells were used for obtaining cell counts every 2 days using an automated cell counter (Logos Biosystems, Inc., Republic of Korea). All experiments were performed in triplicate. The growth rate was estimated between 2 and 8 days using the formula [32] :

$$N_{t}=N_{0}2^{ft}$$

where N_t_, total number of cells; N_0_, initial number of cells; f, growth rate; and t, treatment time. The growth rate values were then used to calculate the doubling time, using the formula [32] :

$$Doubling\;time=\frac{ln(2)}{f}$$

where f, growth rate and ln  =  natural logarithm.

### Soft agar assay for colony formation

Cells (1 × 10^4^) were seeded on 0.3% agar containing 10% FBS underneath 0.6% top agar in a 12-well plate. After a 1-week incubation at 37°C and 5% CO_2_, 1 mL of media was added to the dishes to avoid drying out and to refill sufficient nutrients. Four weeks later, colonies were stained using a 5 mg/mL 3-(4,5-dimethylthiazol-2-yl)-2,5-diphenyltetrazolum (MTT) solution.

### Statistical analysis

Experimental results were statistically evaluated with two-tailed paired student’s t test using Graphpad Prism 7. All tests of significance were set at p < 0.05.

## Results

### Preparation and characterization of five IHOSE cell lines

**Fig 1:**
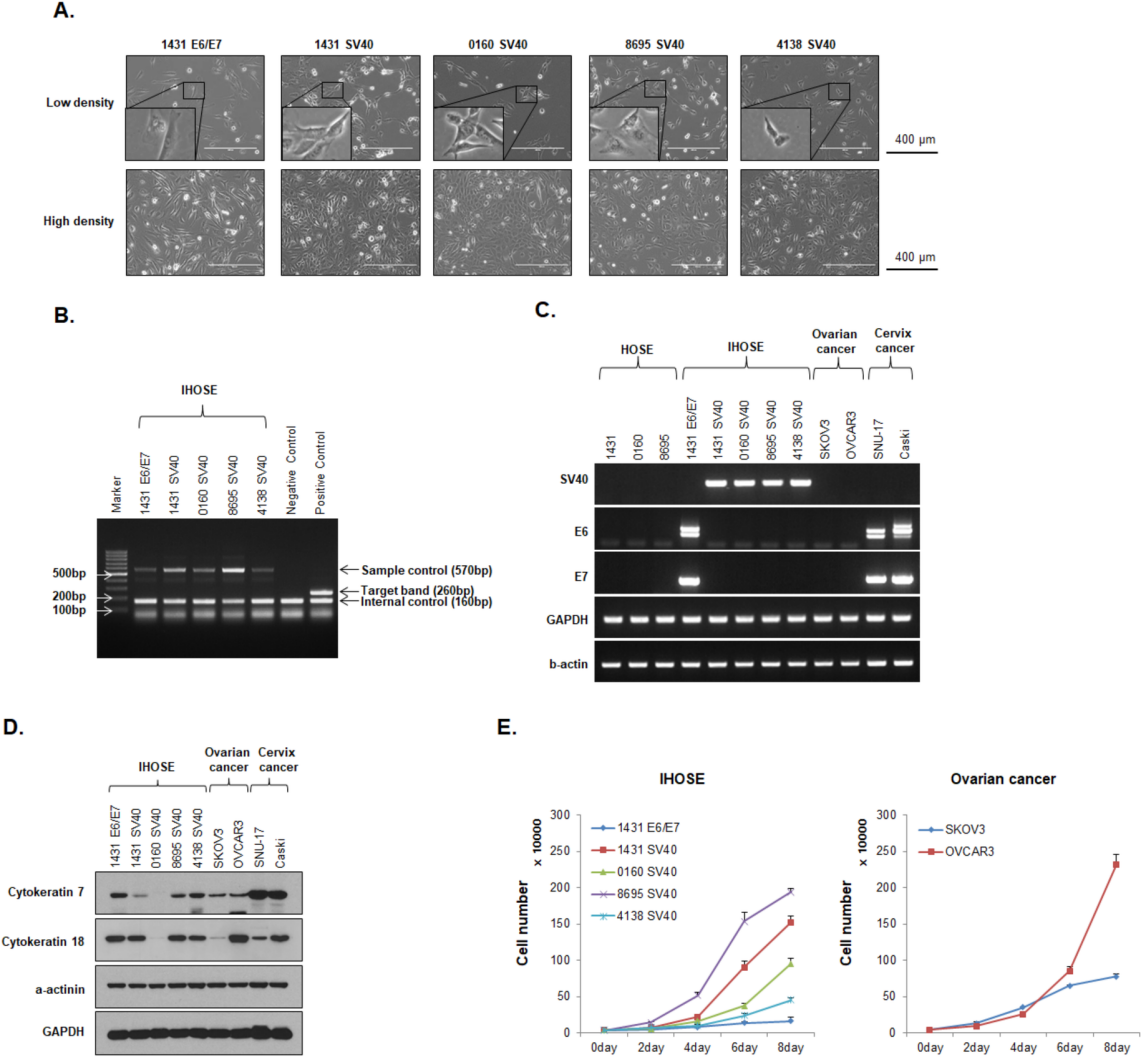
Characterization of five immortalized human ovary surface epithelial (IHOSE) cell lines. **(A)** Representative images showing the morphology of cells from five IHOSE cell lines. SE (Upper panel = Low density of cells; Bottom panel = High density of cells). Scale bar: 400 μm, 100× magnification. **(B)** Mycoplasm contamination testing was performed using 100 ng genomic DNA by RT-PCR. **(C)** The expression levels of SV40 T antigen and HPV E6/E7 protein in IHOSE cells were detected by RT-PCR. β-actin and GAPDH were used as loading controls. **(D)** The expression levels of indicated protein were determined by western blot analysis in the five IHOSE and cancer cell lines. β-actin and GAPDH were used as loading controls. **(E)** Cells (4 × 10^4^) were seeded in a 6-well plate and counted every 2 days up to 8 days. Cell proliferation was determined using an automated cell counter. Results indicate cell number ± SD.

We obtained HOSE cells from four patients without cancer and used a lentivirus to overexpress HPV-E6/E7 and SV40 T antigen in these cells. One of the HOSE cell samples, HOSE-1431, was used to produce two immortalized cell lines that respectively overexpressed HPV-E6/E7 or SV40 T antigen. IHOSE cell lines were established from the other three HOSE cell samples by inducing overexpression of SV40 T antigen only, because HOSE cells expressing HPV-E6/E7 exhibited senescence. Fig 1A shows images of the cells of the five IHOSE cell lines at low or high density. In previous reports, HOSE or IHOSE cells in a monolayer culture exhibited a cobblestone appearance, depending on the conditions of the culture media [33–35], and IHOSE cells infected of hTERT exhibited the co-existence of rod and cobblestone shapes [6]. The five IHOSE exhibited this co-existence with similar a morphology to that of epithelial cells (Fig 1A). Using RT-PCR, we screened for mycoplasma contamination, and confirmed no mycoplasma DNA was present in any of the cell lines (Fig 1B). Further, RT-PCR was used to confirm the successful insertion of the SV40 T antigen and HPV-E6/E7 genes for cell immortalization. All four IHOSE cell lines immortalized by SV40 T antigen expressed the antigen, whereas HPV-E6/E7 was expressed in the other IHOSE cell line. HPV-E6/E7 protein expression was also detected from total RNA samples of SNU-17 and Caski cervical cancer cells as positive controls (Fig 1C). Western blotting was used to detect protein expression of the epithelial cell markers cytokeratin 7 and cytokeratin 18 in IHOSE cells; cytokeratin 18 showed relatively high expression in both IHOSE cells and ovarian cancer cells, whereas cytokeratin 7 was expressed at lower levels in IHOSE cells compared to that found in ovarian cancer cells. However, both cytokeratins were expressed at lower levels in cells from the IHOSE-0160-SV40 cell line and SKOV3 ovarian cancer cells than in the other cells tested (Fig 1D). This difference is consistent with the well-known low expression of cytokeratin genes in SKOV3 cells [36, 37]. From RNA sequencing analysis, we determined that the major expressed cytokeratins in IHOSE and ovarian cancer cells were keratin types 7, 8, 18, and 19. The expression of these cytokeratins in IHOSE cells decreased after immortalization. Especially, the expression level of these keratins was diminished more in IHOSE-0160-SV40 cells than in the other IHOSE cell lines (S1 Table). For the five IHOSE and two ovarian cancer cell lines, the growth rates were measured for up to 8 days. The growth rates of five IHOSE cells differed from each other. 8695-SV40 and 1431-SV40 grew faster than the other IHOSE cells, and 1431 E6/E7 grew the slowest. Compared to the ovarian cancer cell lines, 8695-SV40 and 1431-SV40 grew faster than SKOV3, but their growth was similar with OVCAR3 (Fig 1E and Table 1). Table 1 indicated the doubling time of each cell lines. Genomic DNA was extracted from cells from all five IHOSE cell lines, and DNA fingerprinting was conducted using 16 STR loci. Comparison of STR profiles with the integrated molecular authentication database 2.1 [38] confirmed that all five IHOSE cell lines represent novel cell lines (Table 2). As IHOSE-1431-E6/E7 and IHOSE-1431-SV40 cells share the common HOSE progenitor cell HOSE-1431, identical STR profiling results were obtained for these two cell lines. Consequently, we have established five IHOSE cell lines capable of continuous growth.

**Table 1:** The doubling time of each cell lines.

Cell lines	Doubling Time (h)^a^	Std. Deviation
**1431 E6/E7**	90.20	11.52
**1431 SV40**	35.81	1.55
**0160 SV40**	42.92	1.17
**8695 SV40**	33.67	2.21
**4138 SV40**	52.11	2.47
**SKOV3**	46.02	1.12
**OVCAR3**	33.98	0.67

The doubling time was calculated using results between 2 and 8 days in Fig 1E.

^a^(h) = hour

**Table 2:** STR profiling of five immortalized human ovary surface epithelial cells.

STR Loci	1431 E6/E7	1431 SV40	0160 Myc/SV40	8695 SV40	4138 SV40
**D8S1179**	14, 17	14,17	12, 13	13, 14	10, 16
**D21S11**	29, 33.2	29, 33.2	30	29, 33.2	30
**D7S820**	10, 13	10, 13	12	12	11
**CSF1PO**	10, 12	10, 12	11, 12	9, 10	12, 13
**D3S1358**	15	15	15,18	15, 17	16, 7
**TH01**	7, 9	7, 9	6, 9	9	7, 9
**D13S317**	8, 12	8, 12	10	8, 10	10, 12
**D16S539**	9, 11	9, 11	10, 12	9, 11	9, 13
**D2S1338**	19, 20	19, 20	23, 24	18, 25	23, 24
**D19S433**	13, 14	13, 14	13, 14.2	13, 14.2	13, 14
**Vwa**	18	18	18, 19	16	17, 19
**TPOX**	8, 11	8, 11	8, 9	8	9, 11
**D18S51**	15, 16	15, 16	13, 14	13, 17	13, 14
**Amelogenin**	X	X	X	X	X
**D5S818**	11, 12	11, 12	10, 12	11, 12	10
**FGA**	24, 25	24, 25	21, 23	10, 12	26

### IHOSE cells are genetically closer to HOSE cells than ovarian cancer cells

**Fig 2:**
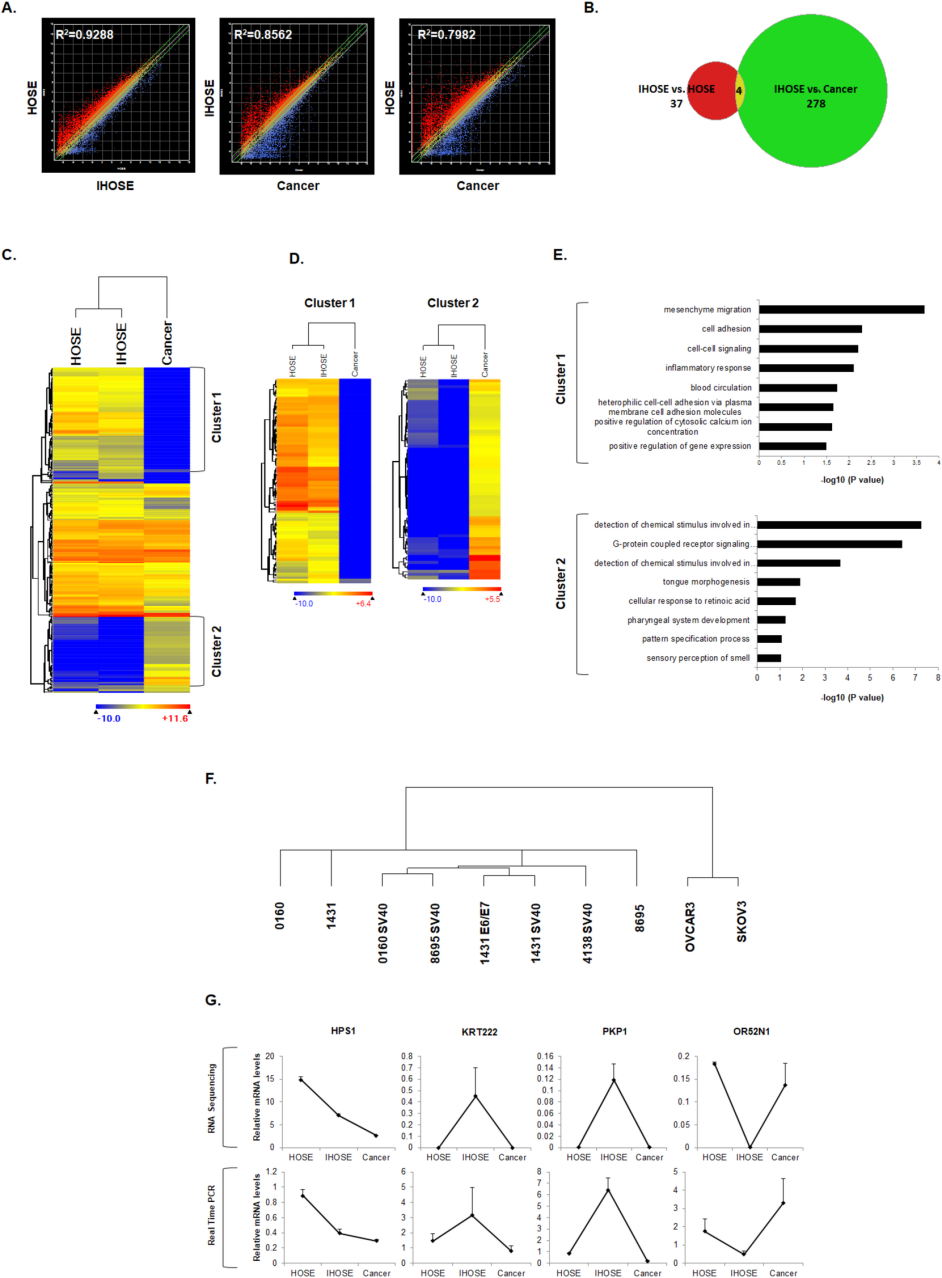
Analysis of differentially expressed genes (DEGs) between IHOSE, HOSE, and ovarian cancer (Cancer) cells using RNA sequencing. **(A)** Scatter plot revealing a linear correlation of gene expression pattern according to comparative analysis in HOSE versus IHOSE, IHOSE versus Cancer, and HOSE versus Cancer. **(B)** Venn diagram showing a common denominator in 319 DEGs based on sorting conditions of 2-fold change, a read-count threshold > 0.1, and p < 0.05. **(C)** Heatmap of 319 DEGs in three analyzed groups. **(D)** Heat map of downregulated genes (Cluster 1) or upregulated genes (Cluster 2) in ovarian cancer compared to HOSE and IHOSE. **(E)** Gene ontology related to biological process ordered according to p-value (–log10). Functional annotation clustering was performed using the DAVID algorithm (Upper panel = Cluster 1; Lower panel = Cluster 2). **(F)** Dendrogram for the clustering of each cell with Euclidean distance metric. **(G)** Validation of RNA-sequencing gene expression levels by real-time PCR for four common DEGs in the three groups. Data are represented as the mean fold change ± SE (Upper panel = RNA sequencing; Lower panel = Real-time PCR).

RNA sequencing was then performed to examine the gene expression patterns in the newly established IHOSE cells in comparison to three of the progenitor HOSE cell lines and two ovarian cancer cell lines. Based on expression data of 22,878 genes (S3 Table), correlation (R^2^) values among the three groups were analyzed through comparison of scatter plots between groups. The correlation in gene expression levels was greater between the IHOSE and HOSE groups (R^2^ = 0.9288) than that found between IHOSE or HOSE and cancer cells (R^2^ = 0.8562 and R^2^ = 0.7982, respectively) (Fig 2A). Gene expression patterns of the 319 differentially expressed genes (DEGs) were obtained based on a signal threshold ≥ 0.1, p ≤ 0.05, false discovery rate of 5%, and two-fold alteration. There were 37 DEGs identified between the IHOSE and HOSE groups, 278 DEGs identified between the IHOSE and cancer groups, and four DEGs among all three groups (Fig 2B and S2 Table). The heatmap representing the expression patterns of these 319 DEGs revealed little difference in gene expression between the HOSE and IHOSE groups, with significant differences evident for the cancer group. This region of significant difference in gene expression was categorized into gene cluster 1 and gene cluster 2 (Fig 2C), reflecting 102 and 67 genes with reduced and enhanced expression in the ovarian cancer group, respectively (Fig 2D). Gene Ontology (GO) analysis of these clusters showed that gene cluster 1 could be divided into 25 GO terms, with "mesenchyme migration" and "cell adhesion" included among the top eight terms. Gene cluster 2 was divided into eight GO terms, with "detection of chemical stimulus involved sensory perception of smell" and "G-protein coupled receptor signaling pathway" ranked highest (Fig 2E). In addition, the dendrogram constructed for each cell type according to the gene expression pattern revealed that IHOSE cells clustered closer to HOSE cells, which were clearly distinguishable from ovarian cancer cells (Fig 2F). To verify the accuracy of the RNA sequencing results, real-time PCR was performed for the four commonly altered genes found in the three groups (Fig 2B). Despite slight differences in the degree of variation among groups, the patterns of increased or decreased gene expression among groups were consistent to those found from RNA sequencing (Fig 2G, top) and real-time PCR results (Fig 2G, bottom). Overall, these findings demonstrate that IHOSE cells have a more similar gene expression pattern to HOSE cells than to ovarian cancer cells, indicating their suitability as control cell lines for cancer research.

### IHOSE cells have no malignant features of cancer cells

**Fig 3:**
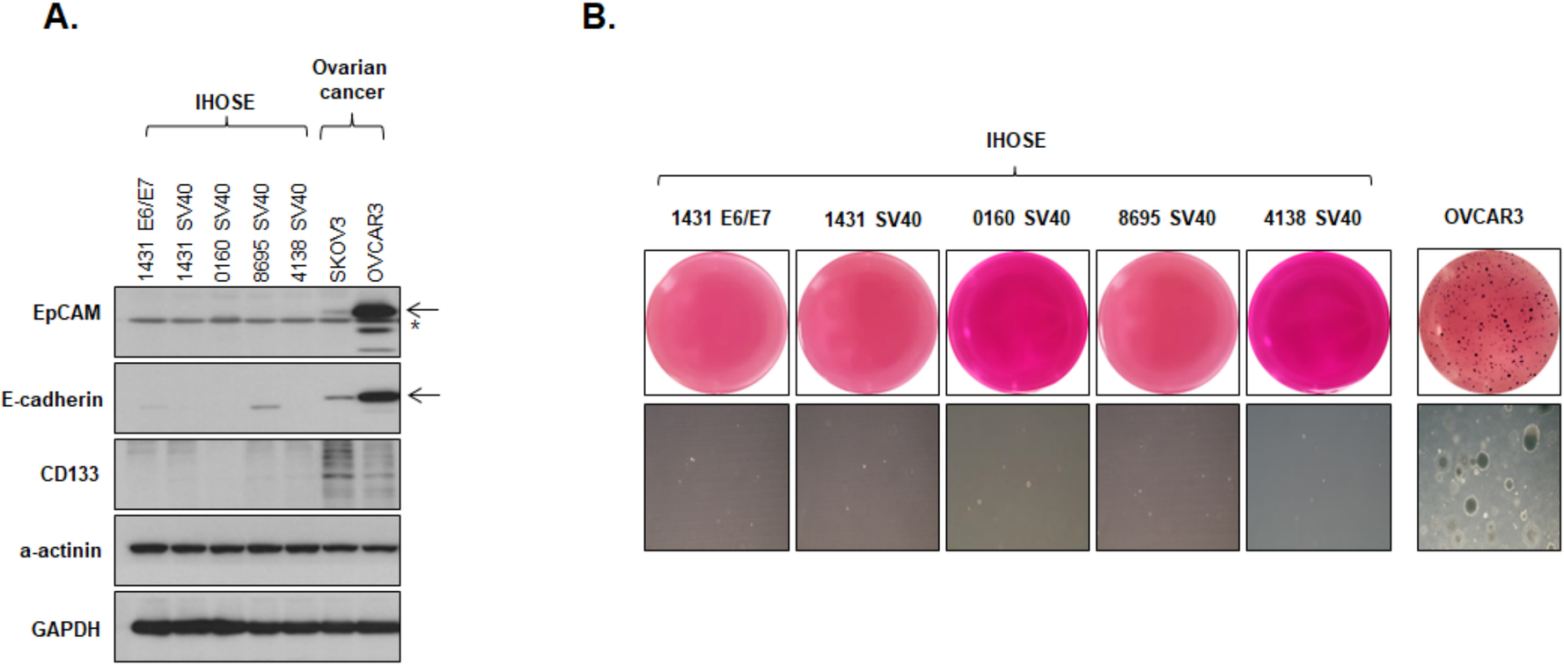
Immortalized human ovary surface epithelial (IHOSE) cell lines have no malignant characteristics. **(A)** The expression levels of indicated protein for the cancer stem markers EpCAM, CD133, and E-cadherin were measured by western blot analysis (Star = non-specific band). **(B)** Soft agar colony formation of 5 IHOSE and OVCAR3 cell lines stained by 5 mg/mL MTT solution. Representative images of wells and colonies (Colonies = 100× magnification).

IHOSE cell lines were established by overexpressing immortalizing proteins to maintain continuous cell division. Since continuous cell division is a prominent feature of cancer cells, we verified whether these IHOSE cells have the malignant features of cancer cells by detecting protein expression levels of cancer stem cell markers using western blotting. We found that two cancer stem cell markers, EpCAM and CD133, were expressed only in ovarian cancer cells, and the expression level of the surface marker E-cadherin was much higher in ovarian cancer cells than in cells from any of the five IHOSE cell lines (Fig 3A). Another feature of cancer cells is able to growth in suspended state. Using the anchorage-independent soft agar assay, we demonstrated that the IHOSE cells did not form colonies as opposed to the ovarian cancer cells that formed colonies (Fig 3B). Therefore, based on the previous results of the RNA sequencing analysis, we showed that the IHOSE cells are more similar to the HOSE cells than the ovarian cancer cell lines. Moreover, the IHOSE cells exhibited low expression of cancer stem cell markers and lack of anchorage-independent growth. Hence, we concluded that the IHOSE cells did not show carcinogenic characteristics of ovarian cancer cells, but they still exhibited the desired property of continuous cell division.

## Discussion

We have been exploring differences in gene expression between the HOSE and ovarian cancer cell lines for more than a decade and have been identifying the clinical implications of various ovarian cancer markers [39–43]. However, culturing of HOSE cells has proven to be a challenge; moreover, the diversity of HOSE pool doesn’t appear to be sufficiently secure. This study was initiated as part of our efforts to directly secure IHOSE cells and to have diversity in the experimental control group, while recognizing that immortalization of HOSE cells, which has previously been conducted in various laboratories, may be one method. Additionally, several papers related to the origin of ovarian cancer have been published in recently. It has been reported that epithelial ovarian cancer cells originate from other organs, and not from the ovaries. Serous tumors have been reported to occur in the epithelial cells of the fallopian tube, and endometrioid and clear cell tumors have been reported to be associated with endometriosis which generally occurs in endometrial tissue due to retrograde menstruation. Thus, the endometrium is also a origin site for these ovarian tumors [29, 31, 44]. Ovarian cancer is a heterogeneous disease that consists of several types of tumors with very different clinicopathological features and behaviors. Therefore, the origin of ovarian cancer is likely to vary. In order to study ovarian cancer, it is necessary to secure normal cells from other pelvic organs in addition to the normal ovarian epithelial cells. Our future goal is to obtain a variety of normal cells that are associated with the development of ovarian cancer, in order to produce a cell line and use it to study ovarian cancer. By the way, a suitable source of carcinogenic tissue and cell lines is essential for research on ovarian cancer to identify candidate biomarkers. However, such studies have been hindered owing to the challenge of obtaining histologically stable samples of HOSE cells as controls. Moreover, even if HOSE cells can be obtained, their use is limited to single short-term experiments because of aging-induced damage that prevents continuous cell division. Thus, the acquisition of HOSE cells and establishment of immortalized cell lines are essential preparative steps to advance the field of ovarian cancer research. Recently, the development of advanced next-generation sequencing technology enables detailed genomic analysis of tumor and cell lines as well as investigation into the key genes and molecular events involved in carcinogenesis [45]. Among these techniques, RNA sequencing is a powerful method for accurately detecting over 10,000 changes at the RNA level using only a small amount of RNA. Thus, we employed an RNA sequencing strategy to compare gene expression profiles of the newly established IHOSE cell lines with each of their progenitor HOSE cells and ovarian cancer cells, demonstrating that IHOSE cells had retained most of the gene expression profiles of their progenitor cells, and were distinct from cancer cells. Notably, the two types of IHOSE cells (1431-E6/E7 and 1431-SV40) established by overexpressing HPV-E6/E7 and SV40 T antigen from the common progenitor HOSE-1431 exhibited identical STR profiles. However, the cell growth rates of these two IHOSE cell lines differed by over two-fold, indicating a strong influence of the immortalization on cell growth properties. Nevertheless, the short Euclidean distance between IHOSE-1431-E6/E7 and IHOSE-1431-SV40 on the dendrogram confirmed that these differences in growth rates were not due to alterations in gene expression during the immortalization process. Further, cells from these two IHOSE cell lines do not seem to have significant differences in overall gene expression compared to cells from the other IHOSE cell lines or progenitor HOSE cells. The consistency between HOSE and IHOSE cells was further confirmed through analysis of DEGs, with clear differences detected from the gene expression patterns of ovarian cancer cells. The gene cluster showing enhanced expression in the cancer group was enriched in the GO term ‘G-protein coupled receptor signaling pathway’, a finding which is consistent with a previous study showing a close association of G-protein-coupled receptors with the development, progression, and metastasis of ovarian cancer [46]. In general, when cell adhesion decreases, cell growth increases along with an increase in migration or invasion [47, 48]. However, the RNA sequencing and GO results are in conflict with this general pattern, demonstrating reduced expression levels of genes involved in cell adhesion and mesenchyme migration in ovarian cancer cells. Cell adhesion molecules are widely known as tumor suppressors because they promote cell adhesion-mediated contact inhibition to reduce cell growth and inhibit tumor dissemination [48]. However, there are also some cell adhesion molecules that can promote cell migration or invasiveness. For example, increasing the expression level of hepaCAM in HepG2 and MCF7 cells results in enhanced cell-extracellular matrix adhesion and cell migration [49]. In addition, overexpression of CEACAM1 in thyroid cancer cells results in increased cell invasion and migration, while CEACAM1 knockdown improved cell growth but decreased cell invasiveness [50]. Based on this background, the present results may imply reduced expression of genes governing migration along with reduced expression of certain cell adhesion molecules in ovarian cancer cells. We further detected a clear difference in the expression of cancer stem cell markers and tumor formation ability between IHOSE cells and ovarian cancer cells. Unfortunately, we were not able to conduct this comparison with additional progenitor HOSE cells because of the limited quantity of cells. We also found that the cancer stem cell markers EpCAM, CD133, and E-cadherin were either expressed at low levels or were absent in IHOSE cells, and none of the five IHOSE cell lines formed colonies in the anchorage-independent soft agar assay. Thus, despite exhibiting immortalization characteristics for the continuous growth of IHOSE cells, these cells do not show the genetic or phenotypic characteristics of cancer cells. Therefore, the established IHOSE cells should be highly useful as a control cell source for ovarian cancer studies, and thus, are expected to become indispensable in basic research on ovarian cancer.

## Supporting information

S1 Table Cytokeratin expression in RNA sequencing data. (XLSX)

S2 Table Gene lists and RNA-sequencing read counts in venn diagram. (XLSX)

S3 Table Whole gene list and read counts (22,878 genes). (XLSX)
